# Combination of native and denaturing PAGE for the detection of protein binding regions in long fragments of genomic DNA

**DOI:** 10.1186/1471-2164-9-272

**Published:** 2008-06-04

**Authors:** Kristel Kaer, Kert Mätlik, Madis Metsis, Mart Speek

**Affiliations:** 1 Department of Gene Technology, Tallinn University of Technology, Akadeemia tee 15, Tallinn 19086, Estonia

## Abstract

**Background:**

In a traditional electrophoresis mobility shift assay (EMSA) a ^32^P-labeled double-stranded DNA oligonucleotide or a restriction fragment bound to a protein is separated from the unbound DNA by polyacrylamide gel electrophoresis (PAGE) in nondenaturing conditions. An extension of this method uses the large population of fragments derived from long genomic regions (approximately 600 kb) for the identification of fragments containing protein binding regions. With this method, genomic DNA is fragmented by restriction enzymes, fragments are amplified by PCR, radiolabeled, incubated with nuclear proteins and the resulting DNA-protein complexes are separated by two-dimensional PAGE. Shifted DNA fragments containing protein binding sites are identified by using additional procedures, i. e. gel elution, PCR amplification, cloning and sequencing. Although the method allows simultaneous analysis of a large population of fragments, it is relatively laborious and can be used to detect only high affinity protein binding sites. Here we propose an alternative and straightforward strategy which is based on a combination of native and denaturing PAGE. This strategy allows the identification of DNA fragments containing low as well as high affinity protein binding regions, derived from genomic DNA (<10 kb) of known sequence.

**Results:**

We have combined an EMSA-based selection step with subsequent denaturing PAGE for the localization of protein binding regions in long (up to10 kb) fragments of genomic DNA. Our strategy consists of the following steps: digestion of genomic DNA with a 4-cutter restriction enzyme (*Alu*I, *Bsu*RI, *Tru*I, etc), separation of low and high molecular weight fractions of resultant DNA fragments, ^32^P-labeling with Klenow polymerase, traditional EMSA, gel elution and identification of the shifted bands (or smear) by denaturing PAGE. The identification of DNA fragments containing protein binding sites is carried out by running the gel-eluted fragments alongside with the full "spectrum" of initial restriction fragments of known size. Here the strategy is used for the identification of protein-binding regions in the 5' region of the rat p75 neurotrophin receptor (*p75NTR*) gene.

**Conclusion:**

The developed strategy is based on a combination of traditional EMSA and denaturing PAGE for the identification of protein binding regions in long fragments of genomic DNA. The identification is straightforward and can be applied to shifted bands corresponding to stable DNA-protein complexes as well as unstable complexes, which undergo dissociation during electrophoresis.

## Background

Electrophoretic mobility shift assay (EMSA), developed by Fried and Crothers [1], and Garner and Revzin [2], is a popular method used for detection of protein-DNA interactions [3]. It is highly sensitive and may be used to obtain qualitative as well as quantitative information in determination of protein binding parameters of various DNA molecules [4-6]. In traditional EMSA, a DNA oligonucleotide or a restriction fragment, generally within the size range of 20–400 bp [7], is radiolabeled and complexed with purified protein or mixture of proteins (nuclear or whole cell extract). This complex is separated from the naked DNA by using polyacrylamide gel electrophoresis (PAGE) under native conditions. Because of the "caging" effect within the gel matrix [8,9], the DNA-protein interactions can be stabilized and the corresponding shifted complexes can be detected as discrete bands. Although in some cases, complexes may dissociate and do not produce detectable shifted bands.

Previously, two similar high-throughput methods were developed for the identification of protein binding regions using a large population of fragments derived from DNAs (plasmids, bacteriophages, bacterial chromosome and human genome fragment) ranging in size from 3 kb to 4,700 kb [10,11]. These methods are relatively laborious because, in addition to the initial two-dimensional PAGE separation step, they require several additional steps (linker addition, PCR amplification, cloning and sequencing) for fragment identification.

Here we describe an alternative and straightforward strategy which is based on a principle of the selection method, known as SELEX [12,13] and uses a combination of native (EMSA) and denaturing PAGE for the identifications of protein binding regions in long (up to 10 kb) fragments of genomic DNA. With this strategy, unique protein binding fragments, which give rise to shifted bands, can be "fished out" and identified. Moreover, DNA fragments which dissociate from the complexes during electrophoresis may be also identifed.

## Methods

### Cells and nuclear extract preparation

Rat pheochromocytoma PC-12 cells (CRL-1721; ATCC, Manassas, VA, USA) [14] were grown in a humidified 5% CO2 incubator at 37°C in Dulbecco's modified Eagle's medium (DMEM) supplemented with 5% fetal bovine serum, 10% horse serum and 100 U/mL of penicillin and streptomycin. All cell culture reagents were purchased from Gibco, Invitrogen, Carlsbad, CA, USA. For nuclear extract preparation, PC-12 cells were washed with 1 × PBS (10 mM Na_2_HPO_4_, 2 mM KH_2_PO_4_, pH 7.4, 137 mM NaCl and 2.7 mM KCl) and lysed in ice-cold buffer containing 10 mM Tris-HCl (pH 8.0), 10 mM NaCl, 1 mM EDTA, 10 mM DTT, 10% glycerol 0.5% NP-40 supplemented with 1 mM PMSF and 1× protease inhibitor cocktail (10 mM Benzamidine, 10 μg/ml Antipain, 2 μg/ml Aprotinin, 10 μg/ml Leupeptin) (Sigma-Aldrich, Bellefonte, PA, USA). Nuclei were pelleted by low speed centrifugation at 350 RCF for 3 min and proteins were extracted with the high salt buffer as described in [15].

### Digestion of genomic DNA and size selection of fragments

A 9.3 kb BamHI fragment of rat genomic DNA derived from the 5' region of neurotrophin receptor (*p75NTR*) gene was cloned into Bluescript KS vector (Stratagene, La Jolla, CA, USA) and digested with restriction enzyme *Bsu*RI or *Alu*I (Fermentas, Vilnius, Lithuania). The fragments obtained were separated on a 1% low gelling temperature agarose (SeaPlaque; FMC, BioProducts Rockland, ME, USA) together with the DNA molecular weight marker (1 kb ladder, Stratagene) run in parallel lane. Two fractions, with approximate sizes of fragments 30–300 bp and 300–700 bp, named low (L) and (H) molecular weight fractions, respectively, were cut out and allowed to diffuse into TE (10 mM Tris-HCl, pH 7.5, 1 mM EDTA; 3 volumes per gel-slice) at 37°C for 8–12 h. These fractions were further concentrated by precipitation with ethanol (2.5 volumes) in the presence of 0.15 M NaOAc (pH 5.5) at room temperature. After centrifugation, the DNA pellets were dissolved in TE at concentration of 100 ng/μl.

### Labeling of DNA fragments

Fragments (400 ng of each fraction) were 3' end-labeled with 20 μCi of [α-^32^P]dCTP (GE Healthcare, Amersham, Buckinghamshire, England) using 1 unit of Klenow polymerase (Fermentas, Vilnius, Lithuania) in a 10 μl reaction volume containing 50 mM Tris-HCl (pH 8.0), 5 mM MgCl_2 _and 10 mM DTT. To favor 3'end-labeling of blunt-ended fragments by 3'-5' exonuclease and 5'-3' polymerase activities of Klenow fragment, the reaction was first incubated in the presence of [α-^32^P]dCTP at 37°C for 10 min. Then all unlabelled dNTPs were added to a final concentration of 0.1 mM for each and the reaction was continued for 25 min. Finally, the reaction was terminated by phenol extraction, ^32^P-labeled fragments were precipitated with 2.5 volumes of ethanol in the presence of 1.5 M NH_4_OAc and dissolved in TE at a concentration of 40 ng/μl.

### EMSA

^32^P-labeled fractions of fragments (80 ng of each) were incubated with PC-12 nuclear extract (1 μg total protein per reaction) in 3 mM Hepes-KOH at pH 7.9, 60 mM KCl, 0.15 mM EDTA, 1.5 mM DTT, 1.5% glycerol, poly (dI-dC) (final concentration 200 ng/μl for low and 400 ng/μl for high molecular fractions, respectively) at room temperature for 20 min. After addition of 20% glycerol (1/5 volume), samples were subjected to electrophoresis on a 3% polyacrylamide gels run with 50 mM Tris-borate buffer, pH 8.3, 1 mM EDTA at room temperature for 2 h. The following electrophoresis reagents were used Tris (ultrapure, Duchefa, Haarlem, The Netherlands), boric acid (ACS grade, AMRESCO, Solon, OH, USA), acrylamide (>99%), N,N'-methylene bisacrylamide (>98%) and EDTA (ACS reagent) from Sigma, St. Louis, MO, USA

Labeled fragments and their shifted complexes with proteins were visualized in wet (preparative) or dried gels by phosphoimaging using Personal Molecular Imager FX system (Bio-Rad Laboratories, Hercules, CA, USA). Shifted bands or zones were located, cut out from the preparative gels and the corresponding labeled fragments were allowed to diffuse into TE as described above. Fragments were concentrated by ethanol precipitation, dissolved in sample buffer containing 90% formamide and 10 mM EDTA and treated at 95°C for 2 min.

### Denaturing PAGE

EMSA-positive shifted fragments, together with ^32^P-labeled L and H molecular weight fractions of fragments and appropriate DNA marker were subjected to electrophoresis on a 40 cm-long 6% denaturing polyacrylamide gel containing 50 mM Tris-borate buffer, pH 8.3, 1 mM EDTA and 7 M urea. After electrophoresis, gel was transferred to the Whatman 3 MM paper, dried and radioactive fragments were visulized by phosphoimaging.

### Sequence analysis and fragment identification

Sequence of the 9.3 kb BamH1 fragment derived from the rat *p75NTR *5' region was obtained from the Genbank^® ^(accession numbers AABR03076992.1 and AABR03076383.1) [16]. Restriction mapping with *Bsu*RI and *Alu*I, and identification of shifted fragments corresponding to protein-DNA complexes were carried out using DNAMAN software package (Restriction analysis) version 4.0 (Lynnon Biosoft, Quebec, Canada).

## Results and Discussion

### A Strategy for the detection of protein binding regions in genomic DNA

**Figure 1:**
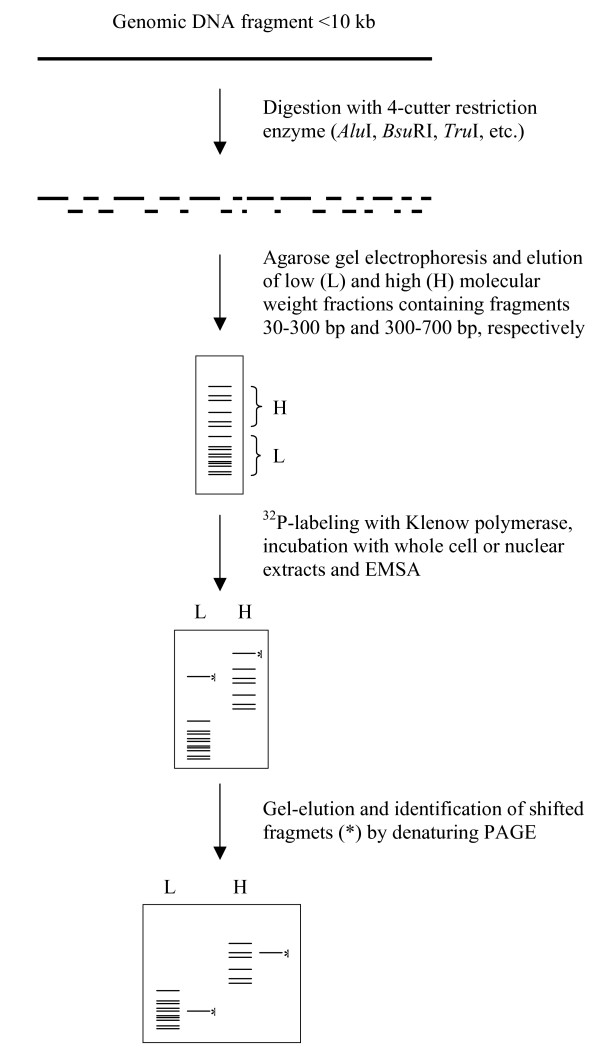
A strategy for the detection of protein binding regions in genomic DNA fragments.

Based on a traditional EMSA and selection of DNA fragments containing protein binding regions from the population of restriction fragments (similar to the SELEX methodology [13]), we have developed a strategy for the detection of protein binding regions in long (<10 kb) fragments of genomic DNA (Fig. 1). This strategy consists of the following steps: fragmentation of genomic DNA with a frequent-cutter restriction enzyme recognizing a 4 bp sequence, isolation of the L and H molecular weight fractions of fragments, ^32^P-labeling with Klenow fragment, EMSA, elution of the shifted fragments and their identification by denaturing PAGE (for details see Methods). The main difference from the traditional EMSA is that instead of using a single labeled oligonucleotide or restriction fragment, a large number of fragments (from 10–50) are used in a single experiment. However, because smaller fragments may give rise to complexes which may be masked by longer fragments, a prior separation of L and H molecular weight fractions of fragments is necessary. After localization and elution of the shifted bands (or smear) from the preparative EMSA-gel, identification of the corresponding protein-bound fragments is carried out by high resolution PAGE. Generally, each shifted DNA fragment can be identified when running it in gel in parallel with the initial pool of labeled fragments of known size (Fig. 1). However, if two fragments of the same size are present among the shifted fragments, their identity may be revealed by the presence or absence of a diagnostic restriction enzyme site, determined from a separate experiment.

### Determination of the protein binding regions in the 5' region of rat neurotrophin receptor (*p75NTR*) gene

**Figure 2:**
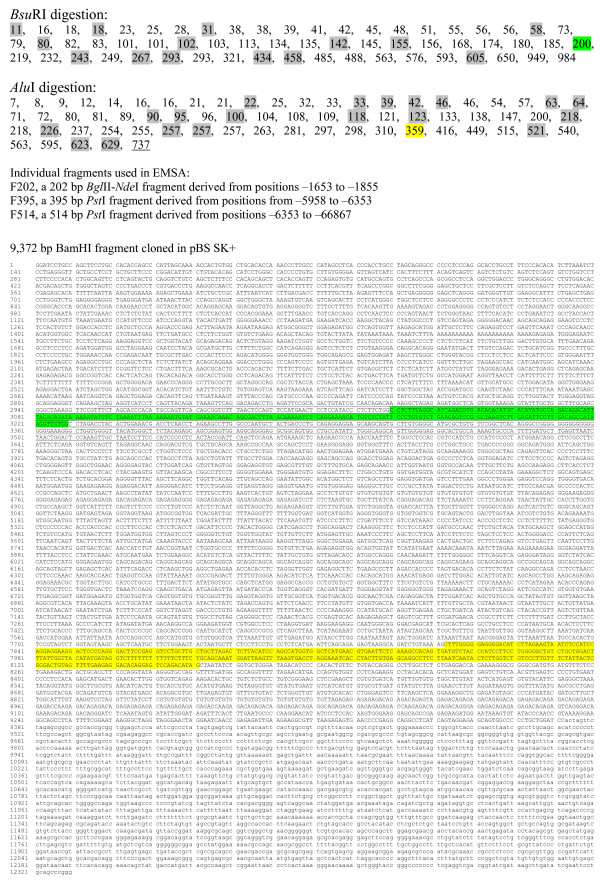
**Fragmentation of the rat *p75NTR *genomic DNA.** A 9,372 bp BamHI fragment derived from 5' region of *p75NTR *(positions from -9,645 to -274) cloned in pBS KS+ (sequence with lower case letters) was digested with *Bsu*RI and *Alu*I. Sequence derived from UCSC Genome Browser on Rat Nov. 2004 Assembly (contigs AABR03076992.1 and AABR03076383.1). Protein binding was detected for fragments marked with green (BsuRI digest) and yellow highlight or underlined (AluI digest). Fragments marked with grey highlight were derived from pBS KS+.

**Figure 3:**
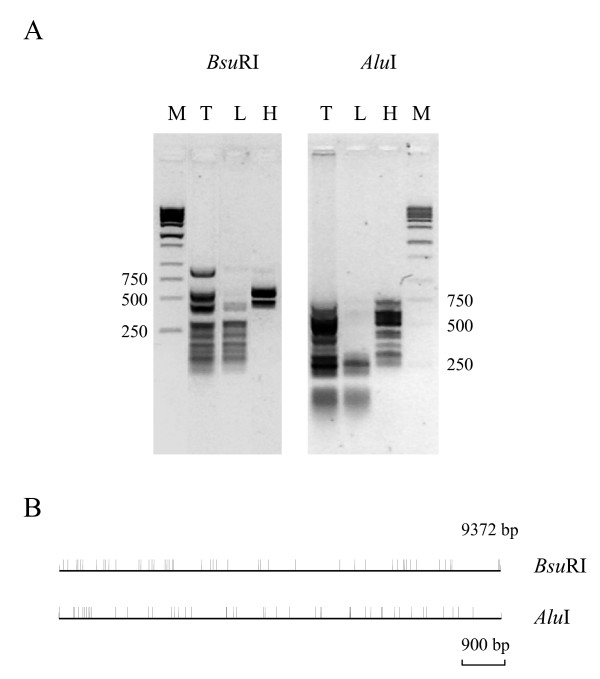
**Digestion of a 9372 bp genomic DNA fragment of the rat *p75NTR *gene (cloned in pBS KS vector) with *Bsu*RI and *Alu*I restriction enzymes.** (A) Agarose gel electrophoresis of total digests (T) and gel-isolated fractions 30–300 bp (L) and 300–700 bp (H). M, 1 kb ladder (Stratagene). (B) The expected digestion pattern generated by sequence analysis with DNAMAN software.

**Figure 4:**
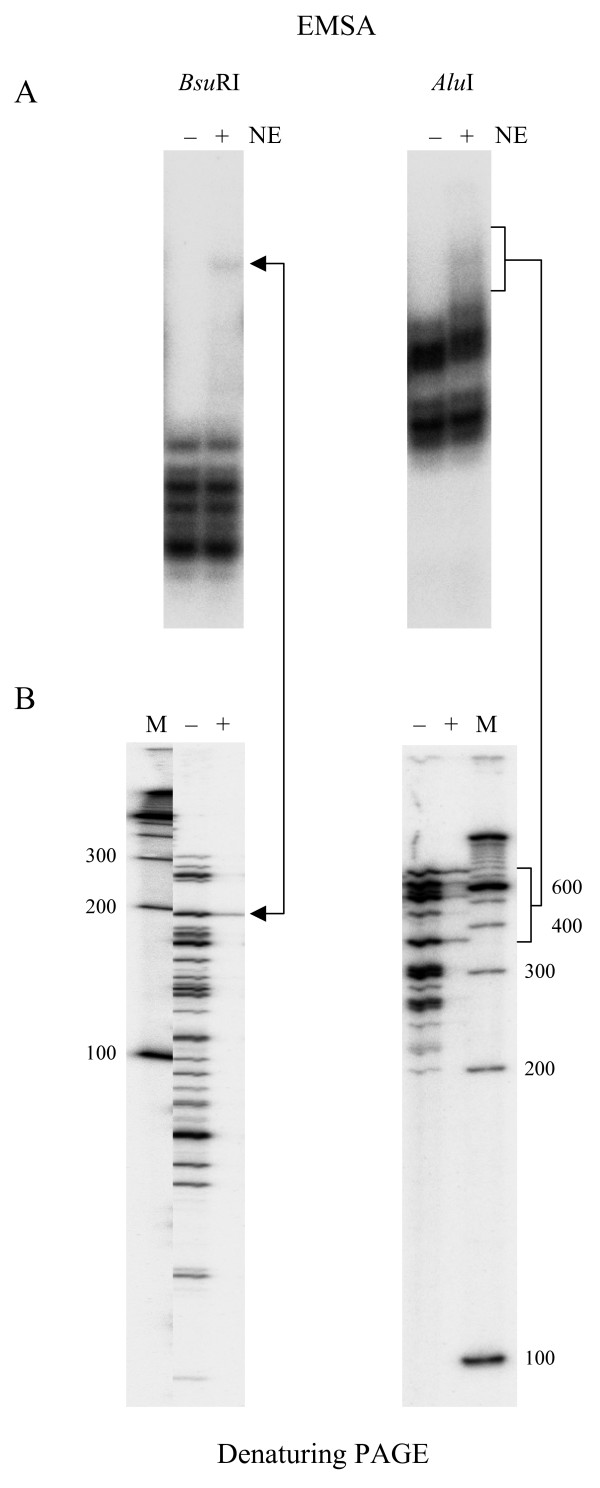
**Identification of the protein binding regions in DNA fragments by a combination of EMSA and denaturing PAGE.** (A) L and H molecular fractions of corresponding *Bsu*RI and *Alu*I fragments were incubated with (lane +) and without (lane -) PC12 nuclear extract (NE) and analysed by EMSA. Direct phosphoimaging of the wet 1 mm-thick gel is shown. (B) Denaturing PAGE of the shifted fragments. Lanes - and + show the pool of fragments used in EMSA and shifted bands (smear), respectively. Connecting lines indicate the bands or smear analyzed on two different gels. M, ^32^P-labeled 100 bp ladder (Gibco-BRL).

To test the strategy, based on a combination of native and denaturing PAGE, we used a 9372 bp fragment corresponding to the nucleotide positions -9,645 to -274 of the upstream (promoter) region of the rat *p75NTR *gene (Fig. 2). Fig. 3A shows the preparation of L and H molecular fragment fractions (30–300 bp and 300–700 bp, respectively). As determined by *in silico *restriction analysis, *Bsu*RI generated 41 fragments in L and 11 in H molecular range. Similarly, *Alu*I generated 43 and 12 fragments in L and H molecular fractions, respectively (Fig. 3B; see also Fig. 2). Representative fractions (L for *Bsu*RI with about 40% coverage and H for *Alu*I with about 70% coverage) were selected for further analysis. Fig. 4 shows the results obtained by using these fractions in EMSA and denaturing PAGE experiments. In the case of *Bsu*RI L fraction, a band with reduced mobilty was identifed as a 200 bp fragment derived from a region -6417 to -6617 bp relative to the transcriptional start site (+1) of the *p75NTR *gene (Fig. 4; see also Fig. 2). However, in the case of *Alu*I H fraction, a smear was observed in EMSA gel (Fig. 4A). A zone covering most of this smear was cut out and analyzed on a denaturing gel (Fig. 4B). This zone contained fragments from 359 to 737 bp in length (Fig. 4B; see also Fig. 2). Comparison of the band intensities between lanes + and -, corresponding to the incubations with and without nuclear proteins, revealed enrichment of two fragments 359 and 737 bp (derived from -1.7 kb and -6.5 kb regions of *p75NTR*; see Fig. 2) suggesting that these fragments contain binding sites for nuclear proteins.

Therefore, this result shows that DNA fragments which dissociate from the complexes during electrophoresis and produce a smear can be identified by denaturing PAGE.

**Figure 5:**
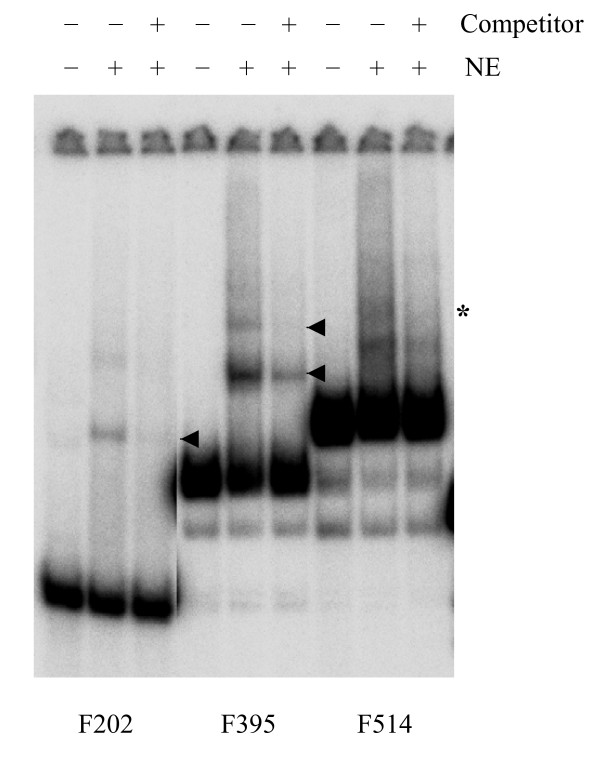
**Confirmation of the protein binding regions with individual DNA fragments.** F202, a 202 bp *Bgl*II-*Nde*I fragment derived from -1.8 kb region partially overlapping with a 359 bp *Alu*I fragment; F395, a 395 bp PstI fragment derived from -6.3 kb region partially overlapping with a 737 bp *Alu*I fragment; F514, a 514 bp *Pst*I fragment derived from -6.8 kb region overlapping with a 200 bp *Bsu*RI fragment (for exact nucleotide positions see Fig. 2). NE, PC12 nuclear extract. A ten-fold excess of the same, but unlabeled fragment was used as a competitor. Arrowheads point at bands resulting from sequence-specific protein-DNA interactions. Note the smearing in case of F514, which suggests dissociation of complex(es) during electrophoresis. The asterisk indicates a weak band, corresponding to a putative specific complex.

**Figure 6:**
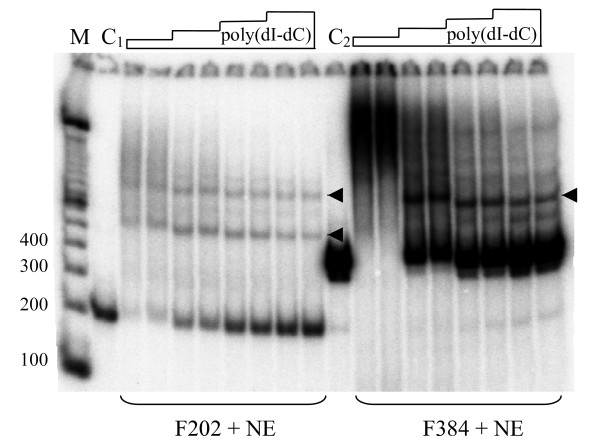
**The effect of the poly (dI-dC) concentration on the mobility shift of restriction fragments.** A poly (dI-dC) concentration range from 100 to 800 μg/ml was tested with 2-fold differences. At each concentration, the incubation of labeled restriction fragment with PC12 nuclear extract (NE) was performed using two different poly (dI-dC) preparations with the average sizes 250 and 500 bp. C_1_, a 202 bp *Bgl*II-*Nde*I fragment and C_2_, a 384 bp *Nde*I-*Mph*I fragment derived from -1.8 kb and -1.6 kb regions of the rat *p75NTR *promoter, respectively. Arrowheads point at the major specific DNA-protein complexes. M, ^32^P-labeled 100 bp ladder (Gibco-BRL).

It is important to note that fragments with sizes greater than 500 bp tend to have nonspecific interactions with proteins and thus may be shifted more frequently than smaller fragments (see also below, Fig. 6). Despite this fact, enrichment of certain fragments in the shifted zone clearly suggests that these may contain regions involved in sequence specific DNA-protein interactions. Consistent with this conclusion, the 737 bp *Alu*I fragment overlapped with the EMSA-positive (or shifted) *Bsu*RI 200 bp fragment detected earlier (Fig. 2). To confirm that the fragments identified here contain binding sites for nuclear proteins, an EMSA was carried out using individual fragments that overlapped with the identified ones (Fig. 5). This experiment showed that all three fragments produced specific shifted products.

### Practical considerations

Previously, two highly similar methods have been developed for the detection of protein binding sites in different DNAs derived from plasmid, bacteriophages (Mu and Lambda), *E. coli *chromosome or human genome [10,11]. These methods are based on two-dimensional PAGE and allow simultaneous analysis of a large collection of fragments (about 2000). However, some DNA-protein interactions may remain undetected because of low affinity or the low concentration of an individual fragment in a large population of fragments [11].

The strategy developed in this study may be suitable for the initial analysis of genomic (promoter) regions extending up to 10 kb in length, revealing protein binding regions that can be further analysed using DNase footprinting [17,18] and promoter activity tests. It is important to note that nonspecific interactions can frequently occur between proteins and longer (>200 bp) DNA fragments used in EMSA. Nonspecific binding may be reduced if higher concentrations of competitor poly (dI-dC) are used (Fig. 6). Therefore, for fragments >200 bp, higher concentrations (up to 1 mg/ml) of competitor must be used. It is recommended that the sufficient concentration of competitor is determined experimentally for each fraction of fragments or individual fragment and nuclear extract.

One of the limitations of traditional EMSA is that some complexes do not withstand the conditions and may produce a smear during electrophoresis. However, as shown here, if the area of the smear is cut out from the gel and the fragments are analyzed, it is possible to identify the fragments undergoing dissociation from the "fragile" complexes (Fig. 4). Alternatively, if the EMSA gel is run at low temperature (+4°C) or at low voltage, it may be possible to stabilize protein DNA interactions and increase the chance of getting shifted fragile complexes [3].

The strategy described here has several advantages over traditional EMSA. It can be performed with a mixture of fragments, thus increasing the length of the DNA fragment that can be analysed in a single experiment. It is based on a simple identification of fragments on a denaturing gel and may be used for the detection of labile interactions (complexes that dissociate during electrophoresis). In principle, the method described by Chernov et al [11] may be also applied to smaller fragments (<10 kb) with an additional improvement, i.e without the need of ligation and PCR. However, it is uncertain whether DNA fragments dissociating from the complexes during electrophoresis can be detected with their method.

**Figure 7:**
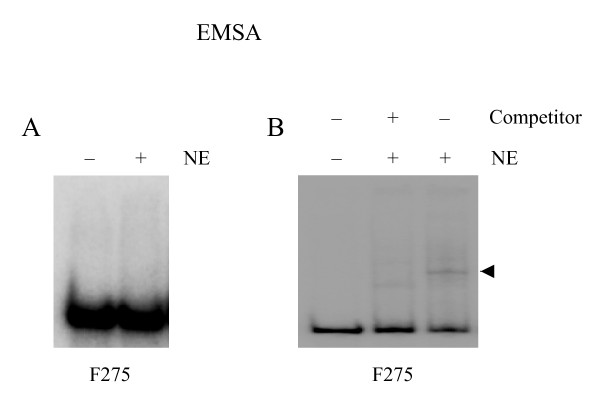
**EMSA experiments with different preparations of DNA fragments.** A 275 bp fragment (F275), derived from L1 antisense promoter [20], was gel isolated by using QIAquick^® ^Gel Extraction Kit (panel A) or gel-diffusion method described here (panel B), radiolabeled and incubated with (+) or without (-) nuclear extract. The presence (+) or absence (-) of competitor, a 10-fold excess of the unlabeled fragment, is shown. Note that specific mobility shift, shown by arrowhead, is visible only in panel B.

Nevertheless, our strategy has also some limitations. Firstly, the 3'-end labeling with 3'-5' exonuclease and polymerase activities of Klenow fragment, is relatively inefficient and produces labeled fragments with different specific activities [19]. Also, fragments with G-C-rich structures at their termini are somewhat resistant to 3'-5-exonuclease of Klenow fragment and thus are difficult to label in some cases. Secondly, some protein binding sites may be digested with the selected restriction enzymes. Thirdly, gel-elution of fragments based on diffusion alone is time consuming. These limitations, however, can be overcome by end-labeling fragments with bacteriophage T4 DNA polymerase, using restriction enzymes with different recognition specificities (e.g., A/T or G/C rich palindromes) and applying quick gel-elution methods [19]. It is worth to mention that our previous experience with two commercial gel extraction kits [QIAquick^® ^Gel Extraction Kit (QIAGEN, Hilden, Germany) and Invisorb Spin DNA Extraction Kit (InViTek, Berlin, Germany)] has shown that certain purified restriction fragments do not produce mobility shifts if isolated at high temperature (50°C), i. e. using a step necessary for dissolving the gel slice (Fig. 7). This is apparently because of the changes in fragment conformation/kinking introduced at high temperature (K. Kaer and M. Speek, unpublished results). Therefore, the diffusion of fragments may be preferred at least for some DNA fragments.

In summary, the strategy developed here may be easily applied to any genomic fragment with known sequence for which protein binding regions are searched. Finally, selection of restriction enzymes with different recognition sequences may be used to expand the fragment coverage and to narrow down the location of protein binding sites.

## Conclusion

We have described a strategy for the detection of protein binding regions in long fragments of genomic DNA. Contrary to the previously described high throughput detection methods [10,11], which can be used to detect high affinity protein binding from the large population of DNA fragments, our strategy uses intermediate (<50) number of fragments and detects low as well as high affinity binding regions. Moreover, as shown here, DNA fragments undergoing dissociation from the DNA-protein complexes during electrophoresis can be identified by this strategy. Our results also stress the importance of testing different poly (dI-dC) concentrations to reduce nonspecific interactions between longer restriction fragments and proteins. We believe that the strategy described here is suitable for the initial analysis of genomic regions (e.g., for searching transcription factor binding sites in promoter regions of genes) and can be complemented by DNase footprinting and promoter activity tests in the later stages of study.

## Authors' contributions

MS and KM designed the strategy, carried out EMSA and PAGE experiments and drafted the manuscript. KK participated in EMSA experiments. MM helped with coordination and revision. All authors read and approved the final manuscript.
